# La salud pública: más allá de la pandemia por COVID-19^1^

**Published:** 2022-03-01

**Authors:** Julián Alfredo Fernández-Niño

**Affiliations:** * Director de Epidemiología y Demografía, Ministerio de Salud y Protección Social Ministerio de Salud y Protección Social

Durante décadas, las discusiones públicas del sector de la salud en Colombia se centraron en tres grandes preocupaciones: la prestación de servicios, el aseguramiento y el financiamiento sectorial. Estos eran los ejes principales del debate político, en tanto que los reclamos de los ciudadanos se extendían a la necesidad de servicios más oportunos y de mayor calidad, así como de una mejor gestión por parte de las empresas aseguradoras de salud. Muchos de esos temas configuraron las propuestas de los candidatos a cargos de elección popular y las agendas de los movimientos sociales en los años recientes.

La salud pública, en cambio, se mencionaba pocas veces excepto en la academia y en los espacios de lucha por el derecho a la salud ^1^. A pesar de su ubicuidad, el propio término era desconocido para muchas personas, o se confundía con otros conceptos. En algunos casos, apenas si se relacionaba con los programas de vacunación, el control de vectores, o las coloquialmente llamadas “charlas de P y P” pero el marco de su acción aparecía desdibujado en la atención clínica curativa que concentraba la opinión pública y política.

La pandemia lo cambió todo. Los ciudadanos y los responsables de las decisiones en el país comenzaron a hablar -un poco más- de salud pública. La Covid-19 puso en evidencia las desigualdades territoriales en las capacidades básicas en ese campo y la necesidad imperante de fortalecer acciones esenciales, como la educación para la salud, la participación comunitaria y la vigilancia epidemiológica, así como de renovadas funciones como la gestión del conocimiento para la toma de decisiones ^2^. Muchas de las acciones impulsadas durante la pandemia, como el autocuidado y el cuidado del otro, requerían de estrategias propias de la salud pública que explican las diferencias observadas en la efectividad territorial. Ahora es necesario que estas acciones y estrategias se consoliden, no solo frente a nuevas emergencias, sino para enfrentar todos los viejos desafíos de la salud pública. Asimismo, el país reconoció el papel de los equipos de vigilancia, que siempre ha sido fundamental, así como el de los muchos técnicos y profesionales en la salud pública, que trabajan en los programas por la salud todos, pero cuyo trabajo efectivo y silencioso no concita titulares.

Probablemente, el 2022 sea el año en que superemos la pandemia de la Covid-19 ^3^, pero las secuelas de la crisis social, ambiental y económica, especialmente la profundización de las desigualdades sociales en salud, seguirán con nosotros mucho tiempo más ^4^.

En mi opinión, y después de algunas reflexiones, tres grandes fuerzas determinan la configuración de la situación de salud en Colombia en la nueva década:

1. la inercia de los cambios demográficos y sociales que anteceden y trascienden la pandemia;
2. la necesidad de formular mejores políticas y programas para la gestión del riesgo individual y colectivo de las principales situaciones de interés en salud pública, y
3. las secuelas multidimensionales de la sindemia, cuyos impactos aún no alcanzamos a dimensionar.

Sobre el primer punto, la inercia de los cambios demográficos y sociales, es claro que el envejecimiento poblacional del país continúa su tendencia ascendente, a pesar del aumento de la mortalidad de adultos mayores durante la pandemia ^5^, con el consecuente incremento de problemas de salud relacionados con el envejecimiento, como el Alzheimer y el Parkinson ^6^.

En cuanto al segundo, políticas y programas para la gestión del riesgo individual y colectivo, es necesario el fortalecimiento de la atención del cáncer y otras enfermedades de alto costo para reducir la mortalidad y mejorar la calidad de vida de los pacientes, lo cual requiere la integración de las rutas de atención en todos los niveles y la reducción al máximo de las demoras y las barreras para el diagnóstico, el tratamiento y los cuidados paliativos.

El tercer punto, las secuelas de la sindemia, hace referencia a las profundas secuelas derivadas de la pauperización de los más pobres y vulnerables, quienes aportaron la mayor proporción de los muertos durante la pandemia (y no solo en Colombia) ^7^ y, que, además, enfrentaron más problemas sociales, como la disminución del ingreso y la pérdida o deterioro del empleo, con el consiguiente estrés financiero y la afectación económica, problemas de grandes consecuencias en la salud mental, aún no del todo medidas. Sin duda, el padecimiento mental, más allá de la enfermedad mental, es uno de los grandes desafíos de la próxima década en el país.

Asimismo, está por evaluarse el impacto de la crisis social y económica de la pandemia en el control de las enfermedades crónicas, pues los recursos se destinaron al manejo de la Covid-19, especialmente durante la fase más crítica de la pandemia, con efectos de largo plazo, especialmente para los más vulnerables. Todo ello se suma a la necesidad de recuperar las coberturas de vacunación contra eventos inmunoprevenibles, retomar los avances en el control de otras infecciones como el dengue y la malaria, y enfrentar problemas reemergentes como la desnutrición infantil, para lo que se requiere mejorar las capacidades de gestión del conocimiento y acción en salud pública local con base en los análisis de la situación de salud ^8^.

Estos problemas, sin embargo, no pueden abordarse únicamente desde el sector de la salud. El marco de acción requerido es necesariamente intersectorial. Problemas como la desnutrición, la obesidad infantil, el tabaquismo, la accidentalidad vial o las violencias, requieren que los demás sectores comprendan que sus acciones también deben articularse cuando el propósito común es el bienestar de la población. Es claro que la Covid-19 y sus secuelas no se superarán de inmediato, pero es necesario que “descoviticemos” la salud pública cooptada por la pandemia y retomemos la agenda previa, así como los desafíos emergentes de la sindemia.

El nuevo Plan Decenal de Salud Pública, 2022-2031, recoge muchos de esos objetivos y aspiraciones comunes. Se trata de una política de Estado, no de gobierno, con una aproximación eminentemente intersectorial, en cuya formulación han participado miles de personas en todo el país y que tiene la tarea, no solo de implementar los aprendizajes y superar las metas pendientes del plan anterior ^9^, sino también, de lidiar con una situación compleja con efectos en múltiples dimensiones que exige un compromiso mayor de las personas con la salud pública, reconociéndola como parte de su vida cotidiana y como el ámbito en el que se fundamentan las posibilidades de una vida de mayor satisfacción y dignidad.

El nuevo plan involucra posturas novedosas, por ejemplo, el reconocimiento de la necesidad del cuidado del ambiente y el afrontamiento del cambio climático como prioridades de salud pública, la determinación de ejes estratégicos transversales de acción en salud pública, y un sistema de vigilancia y evaluación eficiente que sirva realmente para tomar decisiones. Es un plan construido por la ciudadanía y para ella, a partir de las experiencias y anhelos de las personas.

Esta construcción colectiva no es fácil, ya que los debates inherentes a la salud pública son objeto de la lucha social e interesan a múltiples actores e intereses y, por ello, heredan las contradicciones y conflictos en los que se construye el consenso y se reconoce el disenso social; sin embargo, representa una gran oportunidad para construir sobre la convicción de que una vida sana y digna para todos, sin desigualdades injustas y evitables, es posible. Yo quiero pensar que sí.
